# Proteasome α6 Subunit Negatively Regulates the JAK/STAT Pathway and Blood Cell Activation in *Drosophila melanogaster*

**DOI:** 10.3389/fimmu.2021.729631

**Published:** 2021-12-22

**Authors:** Mirva Järvelä-Stölting, Laura Vesala, Matthew K. Maasdorp, Joanna Ciantar, Mika Rämet, Susanna Valanne

**Affiliations:** ^1^ Laboratory of Experimental Immunology, Faculty of Medicine and Health Technology, Tampere University, Tampere, Finland; ^2^ Research Unit for Pediatrics, Pediatric Neurology, Pediatric Surgery, Child Psychiatry, Dermatology, Clinical Genetics, Obstetrics and Gynecology, Otorhinolaryngology and Ophthalmology, Faculty of Medicine, University of Oulu, Oulu, Finland; ^3^ Medical Research Center Oulu, University of Oulu, Oulu, Finland; ^4^ Department of Children and Adolescents, Oulu University Hospital, University of Oulu, Oulu, Finland

**Keywords:** *Drosophila melanogaster*, fruit fly, JAK/STAT pathway, Eye Transformer, the proteasome complex, hemocyte, lamellocyte, RNA interference

## Abstract

JAK/STAT signaling regulates central biological functions such as development, cell differentiation and immune responses. In *Drosophila*, misregulated JAK/STAT signaling in blood cells (hemocytes) induces their aberrant activation. Using mass spectrometry to analyze proteins associated with a negative regulator of the JAK/STAT pathway, and by performing a genome-wide RNAi screen, we identified several components of the proteasome complex as negative regulators of JAK/STAT signaling in *Drosophila*. A selected proteasome component, *Prosα6*, was studied further. In S2 cells, *Prosα6* silencing decreased the amount of the known negative regulator of the pathway, ET, leading to enhanced expression of a JAK/STAT pathway reporter gene. Silencing of *Prosα6 in vivo* resulted in activation of the JAK/STAT pathway, leading to the formation of lamellocytes, a specific hemocyte type indicative of hemocyte activation. This hemocyte phenotype could be partially rescued by simultaneous knockdown of either the *Drosophila* STAT transcription factor, or MAPKK in the JNK-pathway. Our results suggest a role for the proteasome complex components in the JAK/STAT pathway in *Drosophila* blood cells both *in vitro* and *in vivo*.

## Introduction

Regulation of blood cell differentiation and function is a central aspect in immune responses in animals. In addition to the effective activation of the immune cells, controlled silencing of activation is equally important; constant immune cell activity consumes energy and leads to detrimental processes such as autoimmune reactions (1, 2). The Janus kinase/signal transducers and activators of transcription (JAK/STAT) signaling is involved in controlling and regulating biological functions including cell differentiation, developmental processes and immune responses (3). In humans, JAK/STAT is central for blood cell homeostasis, and its aberrant activation can lead to myeloproliferative neoplasms (4, 5). In *Drosophila*, misregulated JAK/STAT signaling in blood cells leads to blood cell activation and formation of tumor-like melanotic blood cell clusters (6–8). The *Drosophila* larval blood cell system consists of three main types of hemocytes: plasmatocytes, lamellocytes and crystal cells (9, 10). Plasmatocytes, the main blood cell type, are round, macrophage-like cells found in all developmental stages in the circulation and in reservoir compartments (11–13). Plasmatocytes are responsible for the phagocytosis of pathogens and apoptotic cells (12, 14, 15). Lamellocytes are a specialized hemocyte type, produced as a response to infection by parasitoid wasps and other pathogens that cannot be phagocytosed (16). They encapsulate the intruder and produce melanin to seal the capsule (17). The third group is crystal cells, which function in the melanization response that is essential for wound healing (18, 19). The activation of several signaling pathways, including the JAK/STAT, Toll, Ras-MAPK and the c-Jun N-terminal kinase (JNK) signaling pathways, is known to induce lamellocyte formation (6, 20).

*Drosophila* JAK/STAT signaling is simpler than its human counterpart, and therefore *Drosophila* offers a prominent model for studying the JAK/STAT pathway, particularly *in vivo* (21). Instead of tens of ligands, four JAKs and seven STATs in humans (22), *Drosophila* only has one JAK (Hopscotch), one STAT (STAT92E) (23, 24) and three cytokine-like ligands (Upd1, Upd2 and Upd3) (25, 26). The signaling pathway is activated when one of the three ligands binds to the receptor Domeless (Dome), a *Drosophila* homolog to the human GP130 receptor (27). Ligand binding leads to the dimerization of Dome and thus, activation of the tyrosine kinase Hopscotch (Hop) (28). Hop activation induces the phosphorylation of the intracellular part of Dome, transphosphorylation of Hop and enables the SH2 domain of the STAT92E transcription factor to attach to the receptor. Hence, Hop phosphorylates STAT92E which dimerizes and translocates to the nucleus where it activates the target genes of the JAK/STAT pathway (29). Similarly to its mammalian counterpart, the *Drosophila* JAK/STAT pathway is involved in several processes including development, stem cell maintenance, immune and stress responses and larval hematopoiesis (25, 30).

Due to its key role in regulating blood cells both in mammals and in insects, it is important to understand the negative and positive regulatory events controlling the activity of the JAK/STAT pathway. We aimed at identifying novel factors that would interact with a known negative regulator of *Drosophila* JAK/STAT signaling, the Eye transformer (ET/CG14225) (31, 32), and therefore participate in the regulation of JAK/STAT activity. In our mass spectrometry study in S2 cells harboring a hemocyte-like identity, we identified several members of the proteasome complex as interactors of ET. Furthermore, we show that silencing *Proteasome α6 subunit in vivo* in *Drosophila* hemocytes induces JAK/STAT activation in them, leading to typical JAK/STAT induced hemocyte phenotypes, the appearance of melanotic nodules and the formation of different types of hemocytes usually present after an immune challenge. Hence, we show that proper functioning of the proteasome complex is crucial for keeping JAK/STAT signaling at bay in hemocytes in healthy animals.

## Materials and Methods

### dsRNA Synthesis

dsRNAs were produced as described previously (31). The primers used for dsRNA synthesis are listed in **Supplementary Table 1**.

### Cell Culture, Transient Transfections and Reporter Assays

The *Drosophila* S2 cells were cultured at 25°C in Schneider’s Insect medium (Sigma, Cat#S9895-1L, USA) containing 0.4 g NaHCO_3_ (Sigma Cat#22350-6, USA) and 0.8 g CaCl_2_ · 2 H_2_O (Sigma S5791-500g, Germany), 10% heat-inactivated Fetal Bovine Serum, FBS (Sigma, Cat#F7524, France) and 50 U/ml Penicillin/Streptomycin (Biochrom AG, Cat#A2213, Berlin).

The S2 cells were transiently transfected using Fugene6 reagent (Roche, Basel, Switzerland) following the manufacturer’s instructions. The JAK/STAT pathway was activated by transfection with either the constitutively active pMT-*hop^Tum-l^* or with the pMT*-upd1-myc* plasmid. To measure the JAK/STAT pathway activation, a *Turandot M-luciferase* (*TotM-luc*) reporter construct, a kind gift from Professor Jean-Luc Imler (University of Strasbourg, France), was used. For studying the effect of RNAi knockdown of selected genes on the expression of ET-V5, S2 cells were transfected with either the pMT-empty or pMT-*Upd1* plasmids together with the pMT-*ET-V5* plasmid and selected dsRNAs. For studying the effect of RNAi knockdown of selected genes on the expression of endogenous *STAT92E*, S2 cells were transfected with the pMT-*Upd1* plasmid for induction of the JAK/STAT pathway. The pMT-empty plasmid was used as a control for uninduced conditions. The protein production from the pMT plasmids was induced 24-48h post transfection by adding CuSO_4_ to each well to a final concentration of 250-500 µM. For Toll and Imd pathway reporter assays, a *Spätzle* or *Imd* overexpressing plasmid was used to induce the pathway and *Drosomycin-luc* or *Attacin-luc* was used as the reporter, respectively (33, 34). In all reporter assays, an Actin5C-β-galactosidase reporter plasmid was used to normalize the results for transfection efficiency and cell numbers as described previously (31). Luciferase and β-galactosidase reporter assays were carried out as previously described in (31).

### Stable Transfection of S2 Cells With Selected Plasmids

The S2 cells were stably transfected with 9.5 µg of each plasmid construct (empty pMT-vector or pMT-*ET-V5* ± pMT-*upd1-myc*) together with 0.5 µg of the coHygro plasmid to enable Hygromycin-B selection of transfected cells. Transfections were carried out on 6-well plates using the Fugene6 transfection reagent (Roche, Basel, Switzerland). Three days post-transfection the transfected cells were selected by adding medium containing 150-300 µg/ml of Hygromycin-B (HyClone Laboratories, Inc. USA). The Hygromycin-B concentration was increased in every passaging up to 300 µg/ml. Under Hygromycin-B selection, only cells carrying the coHygro plasmid survive. From here onwards, the cells were grown under Hygromycin selection.

### Cell Lysis, Protein Extraction and Protein Concentration Measurement

24-48h after the protein production from the pMT-plasmids was induced with CuSO_4_, the cells from three pooled wells, each containing 3 ml of S2 cell suspension, were collected per sample and centrifuged at 5000 x g for 3 min. The supernatants were discarded and 900 µl of lysis buffer (1 x PBS, 1% Igepal CA-630 [Sigma-Aldrich/Merck, Darmstadt, Germany], 0.5% Sodium deoxycholate, 0.1% SDS, 2 mM EDTA, Halt™ Protease and Phosphatase Inhibitor Cocktail, EDTA-free [Thermo Fisher Scientific Inc.]) was added to each sample. The samples were left to lyse on ice for 30 min. The lysates were centrifuged at 16 000 x g for 10 min and the cleared lysates were transferred into new tubes. The protein concentration of each lysate was measured with the Pierce™ BCA protein Assay Kit (Pierce^®^, #23227, USA) according to the manufacturer’s instructions.

### Co-Immunoprecipitation of Protein Complexes With the V5 Antibody, SDS-PAGE and Silver Staining

Putative ET-interaction partners were co-immunoprecipitated from S2 cell protein lysates using Anti-V5 Agarose Affinity Gel (Sigma, #A7345, Israel) according to the manufacturer’s instructions. The following lysates were used: S2 cells (neg. ctrl 1), empty pMT-vector (neg. ctrl 2), pMT-*ET-V5* and pMT-*ET-V5* + pMT-*upd1-myc*. 900 µl of each lysate, containing approximately 2.5 mg of protein, was incubated overnight with the Anti-V5 Agarose Affinity Gel, after which the complexes were washed 6 x 10 minutes with PBS containing protease and phosphatase inhibitor cocktail. All incubations and treatments were carried out at 4°C or on ice. Finally, loading buffer was added on the washed affinity gel containing protein complexes and the samples were boiled to release the proteins. Purified protein complexes were analyzed by SDS-PAGE and silver-staining.

For SDS-PAGE, Novex 10% NuPAGE Bis-Tris (Life Technologies, #NP0301BOX, USA) gels were used. Precision Plus Protein™ Dual Xtra Standard (Bio-Rad, #161-0377, USA) was used as a marker. The electrophoresis was carried out for 45 min using MOPS buffer and NuPAGE Gel program (200 V, 120 mA, 25 W). To stain the protein bands on the gel, the Pierce^®^ Silver Stain for Mass Spectrometry kit (Thermo Scientific, #24600, USA) was used according to the manufacturer’s instructions.

### Mass Spectrometry and Identification of Protein Complex Components

The anti-V5 antibody purified samples were analyzed by mass spectrometry with the help of Tuula Nyman at the University of Helsinki. To identify the proteins in complex with ET, the anti-V5 purified lysates were separated on SDS-PAGE gels and silver stained as described above. The whole sample lanes on SDS-PAGE gel were cut into equal sized pieces after which an in-gel trypsin digestion was performed. The digested peptides were analyzed as described in (35). Flybase Query and Blast tools were used to process the obtained data. The data from the LC-MS/MS was searched against the SwissProt database through ProteinPilot with MASCOT. The following MASCOT search parameters were used: *Drosophila melanogaster* as species, carbamidomethyl modification of cysteine as a fixed modification and oxidation of methionine as a variable modification, trypsin digestion allowing one missed cleavage, 50 ppm mass tolerance and 0.2 Da fragment tolerance for peptides. False discovery rates for the LC-MS/MS data were 1.5-4%.

### SDS-PAGE, Western Blotting and Protein Band Intensity Analysis

For studying the effect of RNAi knockdown of selected genes on the expression of pMT-*ET-V5*, protein lysates were prepared as above and the production of the ET-V5 protein was analyzed by SDS-PAGE and Western blotting. The total protein content of the lysates was set to 50 µg/lane. For SDS-PAGE, Biorad TGX 4-20% mini gels (Bio-Rad, #456-1093, USA) were used. PageRuler™ Prestained protein ladder (Thermo Scientific #26616, Lithuania) was used as a marker. The electrophoretic separation of proteins was carried out for 20-25 min with 300 V using the Tris/Glycine/SDS buffer (Bio-Rad #1610732, USA).

For Western blotting, proteins were transferred from the SDS-PAGE gel either onto 0.45 µm nitrocellulose membrane (Hybond-C extra; Amersham Biosciences, RPN203E, United Kingdom) using the wet transfer technique with an in-house transfer buffer (25 mM Tris, 192 mM glycine, 8% methanol) and the NuPAGE Blot program (25 V, 160 mA, 1 h 15 min), or onto 0.2 µM nitrocellulose membrane using Trans-Blot turbo transfer packs (Bio-Rad, #1704158, USA) and equipment according to the manufacturer’s instructions for TGX mini-gels. The membranes containing the transferred proteins were first incubated with blocking buffer (5% skimmed milk powder in PBS + 0.05% Tween 20 [PBST]) for 2 h at RT or overnight at 4°C. After blocking, the membranes were incubated with primary antibodies in blocking buffer for either 1-2h at RT or overnight at 4°C. The following antibodies were used: anti-V5-HRP conjugate (1:3000, Invitrogen™, Life Technologies, P/N 46-0708), anti-V5 antibody (1:3000, Invitrogen™, Life Technologies P/N 46-0705) and α-tubulin antibody (1:1000, clone DM1A, Sigma-Aldrich). After incubation, the membranes were rinsed three times with PBST and further washed with PBST 4 x 5 min at RT. Goat anti-mouse IgG (H+L) HRP secondary antibody (1:5000, ThermoFisher #G-21040) was used for detecting the mouse monoclonal antibodies (V5 antibody, α-tubulin antibody). Secondary antibody was incubated for 1 h at RT, after which the membranes were washed as before. Immunostained proteins were visualized using an ECL Western Blotting Detection Reagent (either with #RPN2232 by GE Healthcare Amersham™, UK or Westernbright, Advansta, USA) and imaged with the BioRad ChemiDoc™ MP imager using the BioRad ImageLab software (Bio-Rad, California, USA) or by developing the signal on an X-ray film (Fuji Super RX-N film, Japan).

To estimate the amounts of the proteins of interest produced, band intensities on exposed films or imager images were analyzed with the FiJi-ImageJ-64-bit (ver1.51) software. Briefly, images were saved in grayscale in 32-bit mode, after which the bands to be analyzed were selected and their intensities plotted. The intensity of the ET-V5 protein band from the cell lysate was normalized to α-tubulin signal from that lysate. Plotted values were normalized to the control (*GFP* dsRNA-treated) protein band value. Per phenotype, 6-7 replicates were analyzed.

### Fly Stocks

We utilized the GAL4/UAS system (36) to target the expression of transgenes into the fly blood cells, the hemocytes. To visualize larval hemocytes we used the *eaterGFP* (37) and *msnCherry* (38) fluorescent reporters for plasmatocytes and lamellocytes, respectively. These reporters were combined with two hemocyte drivers, *P{Hml-GAL4.Δ}2* [Bloomington *Drosophila* Stock Center, BL #30139 (39)], and P{He-GAL4.Z}*85* [BL #8699 (6)], in order to simultaneously visualize hemocytes and to express transgenic constructs in them, resulting in fly strain *yw,msnF9mo-mCherry,eaterGFP;Hml^Δ^-GAL4;He-GAL4*, hereafter called *mCherry,eaterGFP;Hml* ^Δ^-*GAL4;He-GAL4*. For suppression of gene expression by RNA interference (RNAi), we used the following transgenic lines obtained from the Vienna *Drosophila* Resource Center (VDRC) GD (P-element) and KK (phiC31) RNAi stocks: *UAS-Prosα6^GD^* (VDRC ID #26653), *UAS-Prosα6^KK^* (#100703), *UAS-Stat92E^GD^* (#43867) and *UAS-hep ^GD^* (#2968). The VDRC w^1118^ strains #60100 (denoted as *w^KK^*) and #60000 (denoted as *w^GD^*) were used as genetic background controls for the KK and GD stocks, respectively. The following combination lines were generated: *w; UAS-Prosα6^GD^* (#26653); *UAS-Stat92E^GD^* (#43867) and *UAS-hep^GD^* (#2968); *UAS-Prosα6^GD^* (#26653); +. For testing the *UAS-Prosα6-RNAi* silencing efficiencies, we used a combination of the hemocyte drivers mentioned above, that also contain two inserts of *UAS-eGFP* (*w; HmlΔ -GAL4, UAS-eGFP; He-GAL4, UAS-eGFP*). Of note, to denote the presence of the GAL4/UAS in the figures, we use the symbol “>“.

For detecting the JAK/STAT activity *in vivo*, the *10xStat92E-GFP* [BL #26197 (40)], reporter combined with *He-GAL4* to get *10xSTAT92E-GFP*; *He-GAL4* was used (a gift from Prof. Dan Hultmark). To activate JAK/STAT signaling, the constitutively active form of *Drosophila* JAK, *UAS-hop^Tum-l^* (41) was expressed in hemocytes. 15-20 females of a driver or genetic background control line were crossed with 10 males from transgenic construct lines and kept at 25°C and 12:12 light:dark cycle (LD12:12). Flies were placed daily in fresh vials, and vials with eggs were transferred to 29°C and LD12:12.

### Flow Cytometry Analysis of Larval Hemocytes

Late 3rd instar larvae were washed in water with a brush until clean, placed in a 20 µl drop of 8% bovine serum albumin (BSA) in 1 x phosphate buffered saline (PBS) and carefully ripped open using forceps. The hemolymph was allowed to bleed out and the carcass was removed. The hemolymph sample was pipetted into a 1.5 ml Eppendorf tube with 80 µl of 8% BSA in PBS. 30 µl of the sample was run with a BD Accuri C6 flow cytometer (Becton, Dickinson & Company). Each genotype was analyzed in triplicate (3 x 10 larvae). For the *eaterGFP* and *msnCherry* reporter analysis, a 488 nm 50 mW solid-state laser and 510 +/- 15 nm (FL1, GFP) and 610 +/- 20 nm (FL3, mCherry) optical filters were used to capture the fluorescence signal. GFP-only, mCherry-only and non-fluorescent hemocytes were used to check for the location of these hemocytes and to deduct fluorescence spill over into a wrong channel. By using these reporters, five hemocyte populations can be detected, consisting of single and double positive hemocytes. Following the naming strategy presented in (42), these hemocyte are: plasmatocytes (GFP^high^), activated plasmatocytes (GFP^high^,mCherry^low^), lamelloblasts (GFP^low^), prelamellocytes (GFP^low^,mCherry^low^) and mature lamellocytes (mCherry^high^). The complete procedure of the hemocyte flow cytometry with gating strategies is described in (42).

For the *10xStat92E-GFP* reporter fluorescence measurement, the hemocyte population was separated from the debris based on forward scatter (FSC-A) and side scatter (SSC-A). This population was gated and used in subsequent analyses. A 488 nm 50 mW solid-state laser was used to excite the green fluorescent protein (GFP) and the emission was detected using a 510 +/- 15 nm (FL1) optical filter. Non-fluorescent hemocytes (*w^GD^*) were used as a negative control and hemocytes with *hop^Tum-l^* overexpression (*10xStat92E-GFP;He-GAL4/UAS-hop^Tum-l^*) as a positive control. Hemocytes with a fluorescence intensity above the autofluorescence of the hemocytes in the FL1 channel were considered GFP-positive. A forward scatter area *vs*. height plot (FSC-A *vs*. FSC-H) was used to define the population of round hemocytes, representing the majority of hemocytes in a healthy *Drosophila* larva, the plasmatocytes. These hemocytes were gated, and the GFP fluorescence intensity was measured for all hemocytes gated in the FSC-SSC plot and separately only for the hemocytes falling into the plasmatocyte gate.

### Hemocyte Imaging

3^rd^ instar larvae were bled in a drop of 1% BSA in PBS on a 12-well glass slide, pooling hemocytes from three larvae per well. Hemocytes were allowed to attach and spread on the slide for 50 min, after which they were fixed for ten minutes using 20 µl of 3.7% paraformaldehyde. After fixation, wells were washed three times using cold 1 x PBS and permeabilized for 5 min using 0.1% Triton-X100. After washing three times using cold 1 x PBS, cells were incubated for 30 min with 20 µl of Alexa Fluor 680 -conjugated Phalloidin (Invitrogen), in a 1:50 dilution in 1% BSA in PBS, to stain filamentous actin. The wells were washed, and the samples mounted using ProLong Gold antifade reagent with DAPI (ThermoFisher Scientific) and Zeiss coverslips (thickness no. 1 ½, 18 x 18 mm). Slides were left to cure overnight at room temperature protected from light and imaged with Zeiss LSM 780 laser scanning confocal microscope using a 40x oil immersion objective at several random locations on the next day. 405 nm, 488 nm and 628 nm lasers were used to excite DAPI, GFP and AlexaFluor 688 nm, respectively, and emission was collected at 410-488 nm, 490-544 nm and 661-759 nm. Multiple layers were imaged in Z-plane at 0.36 µm intervals. ImageJ version 2.1.0/1.53 c was utilized to create stacked images. ImageJ and Adobe Photoshop (release 22.5.1) were used to enhance the fluorescence signal for better visibility, keeping modifications constant across the images.

### Pupation and Eclosion Success and Larval and Pupal Melanotic Nodules

The effect of silencing *Prosα6* in hemocytes on the number of successfully pupated and eclosed animals was assessed in *msnCherry,eaterGFP;Hml^Δ^-GAL4;He*-*GAL4* flies crossed with *UAS-Prosα6^GD^*, *UAS-Prosα6^KK^* and *w^GD^* flies. Three to four replicate crosses of each genotype were made, and adults were transferred to fresh vials on three subsequent days. Eggs were counted from each vial and the vials were monitored daily for pupated/eclosed animals. At the same time, the number of pupae with melanotic nodules was recorded. Examples of pupae with melanotic nodules were imaged with a Nikon DS-Fi2 camera attached to a Nikon SMZ745T microscope and a Nikon DS-L3 camera control unit.

The prevalence of larval melanotic nodules was assessed for the same genotypes in three replicate crosses (100 animals each) by inspecting the larvae under a stereomicroscope. Example images of larvae (20x magnification) were taken with a Deltapix Invenio 10EIII camera (DeltaPix, Denmark) attached to a Nikon SMZ745T stereomicroscope using the DeltaPix InSight software.

### Lifespan Experiment

The effect of silencing *Prosα6* in hemocytes on the viability of the flies was assessed by monitoring the lifespan of the flies compared to controls. *UAS-Prosα6^GD^* and *UAS-Prosα6^KK^* RNAi lines were crossed with the *msnCherry,eaterGFP;Hml^Δ^*-GAL4*; He*-GAL4 flies (described above) at 25°C. As a control, *msnCherry,eaterGFP;Hml^Δ^*-GAL4*; He*-GAL4 flies were crossed with *w^GD^* flies. The fly eggs were transferred to 29°C, and this temperature was used for the entire lifespan of the flies. Every 2-3 days the number of living flies was recorded, and the flies were transferred to fresh food.

### RNA Extraction and qRT-PCR from S2 Cells and Hemocytes

Expression levels of selected genes in S2 cells and hemocytes were measured by quantitative reverse transcriptase PCR (qRT-PCR) from extracted RNA. For monitoring expression levels in S2 cells, the cells were grown on 24-well plates, treated with selected dsRNAs and transfected with selected plasmids as described above. For studying expression levels in hemocytes *in vivo*, hemocytes from 50 dissected larvae were collected for each replicate; three biological replicates from three independent crosses were used per genotype. The RNA extraction both from hemocytes and cultured cells was performed using the TRI reagent (MRC, Thermo Fisher Scientific). S2 cells were harvested from culture plates by pipetting and centrifugation (5000 x g, 3 min), after which cells were homogenized and lysed in TRI reagent by pipetting up and down at least 10 times. Extraction from hemocytes was started by adding TRI reagent onto the frozen hemocyte pellet, and cells were homogenized and lysed by pipetting up and down at least 10 times. Thereafter the extraction was performed according to manufacturer’s instructions. qRT-PCR was carried out from extracted RNAs (30-40 ng RNA/sample) using the iTaq Universal SYBR Green Onestep kit (Bio-Rad, Hercules, CA). The expression values obtained for selected genes were normalized to the expression of a gene encoding for ribosomal protein L32 (*RpL32*). The primers used are listed in **Supplementary Table 1**.

### Statistics

Statistical analyses of luciferase assay measurements, qRT-PCR results and protein band intensity analyses were carried out using the two-tailed Student t-test for two arrays assuming equal variances. Statistical analyses of fly life span experiments were carried out with the log-rank (Mantel-Cox) test using Prism 6 (GraphPad) software. The difference was considered statistically significant if the p-value was < 0.05. Proportional data were analyzed using a Generalized linear model (GLM) with binomial distribution and combined with Tukey’s *post-hoc* test for pairwise comparisons. The data on hemocyte numbers were analyzed using a negative binomial GLM followed by pairwise comparisons of groups using estimated marginal means. A Kruskal-Wallis rank sum test combined with Dunn’s *post hoc* test was applied to GFP intensity comparisons in **Figure 3D**. R version 4.0.4 was used to perform GLM and Kruskal-Wallis rank sum tests.

## Results

### Potential Interaction Partners of Eye Transformer (ET) Analyzed by Mass Spectrometry

In a previous study, we used a large-scale RNAi screen in *Drosophila* S2 cells to discover genes important for the *Drosophila* JAK/STAT pathway (31). In the screen we identified Eye transformer (ET) as a negative regulator of the JAK/STAT pathway (31). To screen for putative ET interactors, we created a *Drosophila* S2 cell-line stably overexpressing the ET protein with a V5 tag (ET-V5), and affinity-purified all the proteins in the ET complex with the V5 antibody. In **Supplementary Figure 1A**, it is shown that the ET-V5 signal can be detected with the V5-antibody (α-V5) in the lysates from ET-V5-expressing S2 cells. Since this control experiment showed that ET-V5 is successfully captured with this method, we next affinity purified proteins in complex with ET, separated them using SDS-PAGE and visualized the proteins by silver staining (**Supplementary Figure 1B**). Each line was cut, digested with trypsin and the protein composition was determined by mass spectrometry.

The potential ET interaction partners were studied in two situations, either with or without overexpression of the Dome receptor ligand *upd1*. When the ligand is present, the JAK/STAT pathway is activated, whereas without the ligand, the pathway stays inactive. In addition, untreated S2 cells and S2 cells transfected with an empty pMT vector were used as negative controls. After proteins that were also found in the negative controls were excluded, we identified in total 173 *Drosophila melanogaster* proteins in complex with ET under the conditions where the JAK/STAT pathway was inactive (ET-V5 alone) and 175 proteins when the ligand Upd1 was present (*ET-V5* + *Upd1-myc*). Out of these, 136 proteins were found in both situations (ET with and without Upd1 induction). The mass spectrometry raw data, including putative candidates for JAK/STAT pathway regulation, is shown in **Supplementary Table 2**.

### The Proteasome Complex Components Negatively Regulate JAK/STAT Pathway Activation in S2 Cells

**Figure 1:**
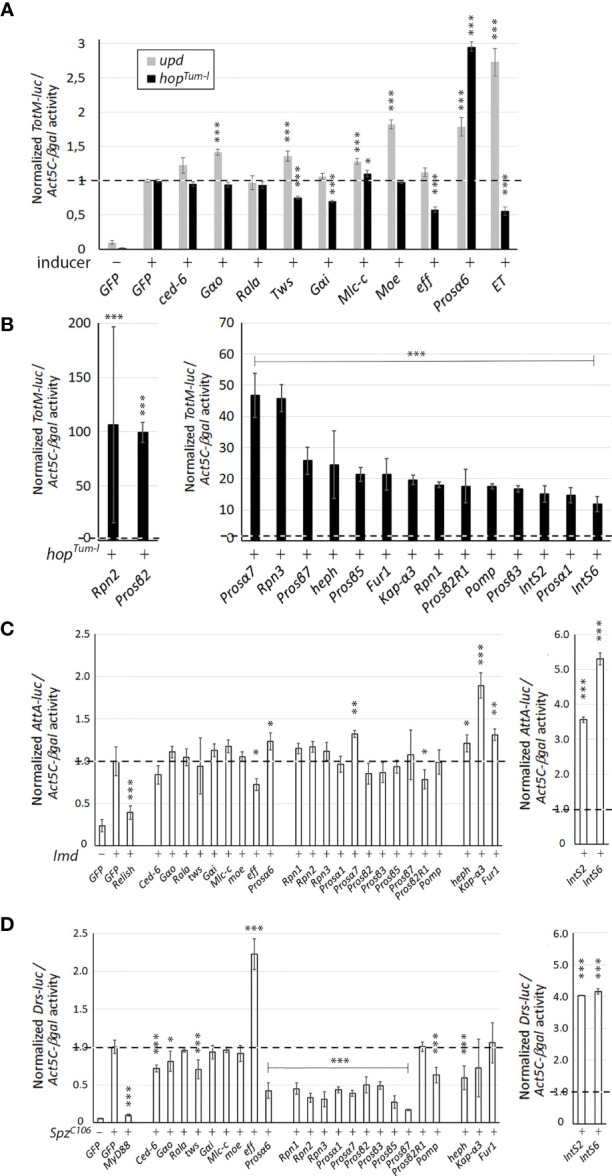
The effect of RNAi of the putative JAK/STAT pathway regulators on the *Drosophila* immunity pathways. **(A)** The effect of RNAi of putative ET interaction partners identified in the mass spectrometry study on the activation of the JAK/STAT pathway. The JAK/STAT pathway was induced by overexpression of *Upd1* (gray bars) or *hop^Tum-l^* (black bars) and the activation of the *TotM-luc* reporter was measured. **(B)** RNAi of the negative regulator candidates from the genome-wide screen in S2 cells causes hyperactivation of the pathway induced by overexpression of *hop^Tum-l^*. **(C)** The effect of RNAi against the candidate genes identified in A and B on the Imd-induced Imd pathway reporter (*AttA-luc*) activity. **(D)** The effect of RNAi against the candidate genes identified in A and B on the Spz^C106^-induced Toll pathway reporter (*Drs-luc*) activity. In all reporter assays, n=4 per dsRNA treatment, and luciferase reporter values were normalized to the values of the Act5C-βgal reporter activity. The relative reporter activity value of cells with an activated pathway treated with the negative dsRNA control (GFP) is set to 1. Statistical analyses were carried out using Student t test for two samples assuming equal variances. *p, 0.05, **p, 0.01, ***p, 0.001. n.s., not significant.

The mass spectrometry screen identified in total 212 unique putative interaction partners of ET (**Supplementary Table 2**). These genes/proteins were compared to the Kallio & coworker’s screen for JAK/STAT components (31) as well as other literature, to search for links to the JAK/STAT pathway, including immunity, hematopoiesis or stress response (25). In total, nine genes were selected for further study: four gene products where the interaction was detected with ET alone, three gene products where the interaction with ET was detected upon Upd1 induction and two gene products where the interaction was detected with ET both with and without Upd1 induction. The selected genes are listed in **Table 1**, with a description of the gene function deduced from the www.flybase.org gene pages. To assess if the association of ET with these nine gene products signifies a regulatory effect on the JAK/STAT pathway, we carried out a JAK/STAT pathway reporter assay in S2 cells with dsRNAs targeting the genes (**Figure 1A**). As shown in **Figure 1A**, induction with overexpression of either *upd1* (gray bars) or *hop^Tum-l^* (black bars) causes an activation of the JAK/STAT pathway, as assessed by the production of the luciferase signal from the JAK/STAT target gene luciferase reporter construct *Turandot M-luciferase* (*TotM-luc*). *GFP* dsRNA was used as a negative control in both assays. Knockdown of *ET* was used as a positive control in measuring the Upd1-induced *TotM-luc* activity, but as ET is upstream of Hop, its knockdown does not have an increasing effect on Hop^Tum-l^ -induced *TotM-luc* activity, but instead, a decreasing effect, as discussed e.g., in (31). Of the nine RNAi treatments tested, only *Proteasome α6 subunit* (*Prosα6*) causes hyperactivation of the JAK/STAT pathway upon *hop^Tum-l^* induction. Prosα6 is a component of the core particle of the proteasome complex [see below (43)]. The Upd1-induced *TotM-luc* signal is enhanced when *G protein α o subunit* (*Gαo)*, *Myosin light chain cytoplasmic* (*Mlc-c)*, *Moesin* (*Moe)*, *Prosα6* or *Twins* (*tws)* are knocked down by targeted RNAi.

**Table 1:** Selected putative ET interaction partners from the mass spectrometry study.

CG number	Gene name	Symbol	Description
***Interaction detected with ET alone:***			
*CG11804*	*ced-6*	*ced-6*	intracellular adaptor protein, involved in signal transduction (phagocytosis of apoptotic cells)
*CG2204*	*G protein α o subunit*	*Gαo*	involved in signaling by a variety of GPCRs
*CG2849*	*Ras-like protein A*	*Rala*	GTPase known to regulate Notch, JAK/STAT and JNK signaling pathways
*CG6235*	*Twins*	*tws*	regulatory subunit of protein phosphatase 2A
***Interaction detected with ET upon Upd induction:***			
*CG10060*	*G protein α i subunit*	*Gαi*	G protein α subunit, sequence homology to mammalian Giα that inhibits adenylate cyclase activity
*CG3201*	*Myosin light chain cytoplasmic*	*Mlc-c*	subunit of the myosin complex, involved in actin filament-based movement
*CG10701*	*Moesin*	*Moe*	involved in cortical cytoskeleton stability, regulates the products of *crb* and *Rho1*
***Interaction detected with ET both with and without Upd induction:***			
*CG7425*	*Effete*	*eff*	conserved class I E2 ubiquitin-conjugating enzyme, protein ubiquitination and degradation pathway
*CG4904*	*Proteasome α6 subunit*	*Prosα6*	Proteasome 35kD subunit, endopeptidase activity, orthologous to human PSMA1 (proteasome 20S subunit alpha 1).

We have previously carried out a genome-wide JAK/STAT pathway RNAi screen (31), and when analyzing unpublished putative negative regulators of the pathway found in the screen, we identified 16 genes whose RNAi caused more than a 10-fold hyperactivation of the JAK/STAT pathway (**Table 2** and **Figure 1B**). Of these, 69% (11/16) had a described function in the proteasome pathway. The five non-proteasome-related putative negative regulator genes, are *hephaestus* (*heph*), *karyopherin α3* (*Kap-α3*), *Furin1* (*Fur1*), *Integrator 2* (*IntS2*) and *Integrator 6* (*IntS6*).

**Table 2:** JAK/STAT pathway negative regulators from the genome-wide RNAi screen (cut off FC = 10), grouped by function.

CG number	Name	Symbol	Description	Human ortholog
**Members of the 26S proteasome complex, components of the 19S regulatory particle**				
CG7762	*Regulatory particle non-ATPase 1*	*Rpn1*	zinc ion binding, enzyme regulator activity	
CG11888	*Regulatory particle non-ATPase 2*	*Rpn2*	zinc ion binding, enzyme regulator activity	PSMD1 ^(1^
CG10484/CG42641	*Regulatory particle non-ATPase 3*	*Rpn3*	zinc ion binding, enzyme regulator activity	PSMD3 ^(1^
**Members of the 26S proteasome complex, components of the 20S core particle, α subunits (outer rings)**				
CG18495	*Proteasome α1 subunit*	*Prosα1*	predicted endopeptidase activity	PSMA6 ^(2^
CG1519	*Proteasome α7 subunit*	*Prosα7*	predicted endopeptidase activity	PSMA3 ^(2^
**Members of the 26S proteasome complex, components of the 20S core particle, β subunits (inner rings)**				
CG3329	*Proteasome β2 subunit*	*Prosβ2*	predicted endopeptidase activity	PSMB7 ^(3^
CG11981	*Proteasome β3 subunit*	*Prosβ3*	predicted endopeptidase activity	PSMB3 ^(3^
CG12323	*Proteasome β5 subunit*	*Prosβ5*	predicted endopeptidase activity	PSMB5/PSMB8 ^(3^
CG12000	*Proteasome β7 subunit*	*Prosβ7*	predicted endopeptidase activity	PSMB4 ^(3^
CG18341	*Proteasome β2 subunit-related 1*	*Prosβ2R1*	predicted endopeptidase activity	PSMB7 ^(3^
**Other, proteasome-related**				
CG9324	*Pomp*	*Pomp*	A chaperone protein, incorporation of 20S core particle β subunits	POMP ^(4^
**Other, not proteasome-related**				
CG31000	*hephaestus*	*heph*	nucleo-cytoplasmic shuttling protein, involved in Notch signaling regul.	PTBP1/PTBP2/PTBP3 ^(5^
CG9423	*karyopherin-alpha3*	*Kap-α3*	Notch binding, myosin binding, prot. Nucl. Import	KPNA3/KPNA4 ^(6^
CG10772	*Furin1*	*Fur1*	serine-type endopeptidase activity, plasma membrane	FURIN
CG8211	*Integrator 2*	*IntS2*	component of the Integrator complex	INTS2 ^(7^
CG3125	*Integrator 6*	*IntS6*	component of the Integrator complex	INTS6/INTS6L ^(7^

(1 PSMD = proteasome 26S subunit non-ATPase.

(2 PSMA = proteasome 20S subunit alpha.

(3 PSMB = proteasome 20S subunit beta.

(4 POMP = proteasome maturation protein.

(5 PTBP = polypyrimidine tract binding protein.

(6 KPNA = karyopherin subunit alpha.

(7 INTS = integrator complex subunit.

The proteasome is a large protein complex consisting of a 20S core particle capped by 19S regulatory particles (43). Among the seven alpha rings of the proteasome core particle, RNAi-mediated silencing of *Prosα1*, *Prosα6* and *Prosα7* caused hyperactivation of the JAK/STAT pathway as did silencing of four of the seven beta ring sub-particles and one related gene, namely *Prosβ2*, *Prosβ3*, *Prosβ5*, *Prosβ7* and *Prosβ2R1*. Moreover, silencing of the *Drosophila* 19S regulatory particle genes *Rpn1*, *Rpn2* and *Rpn3*, as well as proteasome-related chaperone protein *Pomp*, had similar effects (**Table 2** and **Figure 1B**). In conclusion, RNAi-mediated silencing of the expression of proteasome genes causes hyperactivation of the JAK/STAT pathway in S2 cells [**Table 2** and **Figure 1B** (44)].

### The Effect of RNAi-Mediated Silencing of the Putative JAK/STAT Pathway Regulators on the Activity of the Imd and Toll Pathways

The total of twenty-five genes from the study of ET interaction partners (**Table 1** and **Figure 1A**) and from the Kallio & coworkers’ RNAi screen for negative regulators of the JAK/STAT pathway (**Table 2** and **Figure 1B** (31), were selected for further study. To investigate if these genes act in the regulation of other immune-related pathways besides JAK/STAT, we tested if their silencing affects the activation of the Imd or Toll pathways using reporter assays. To investigate effects on the Imd pathway, the pathway was induced by transfecting S2 cells with the Imd plasmid and relevant dsRNAs. *GFP* was used as a negative control and *Relish* dsRNA as a positive control. As shown in **Figure 1C**, most of the genes studied do not regulate the Imd pathway; however, knockdown of *effete* (*eff*) and *Prosβ2R1* leads to a slight decrease in pathway activation compared to *GFP*. Effete, the *Drosophila* homolog of the Ubc5 E2-ubiquitin conjugating enzyme, has previously been shown to be needed for Imd ubiquitination (45). Knockdown of *Prosα6*, *Prosα7*, *heph* and *Fur1* leads to a slight elevation in the Imd pathway activation, whereas when *Kap-α3*, *IntS2*, and *IntS6* are silenced, the pathway is markedly upregulated (**Figure 1C**).

To investigate the effects of silencing of the selected 25 genes on the Toll pathway, the pathway was induced by overexpression of the *Spz^C106^* plasmid. A *MyD88* dsRNA treatment was used as a positive and *GFP* as a negative control of the pathway. As shown in **Figure 1D**, the silencing of several genes resulted in a reduction in Toll pathway activity, including mainly components of the proteasome: *Rpn*-genes, *Pomp*, *Prosα*- and *Prosβ*-genes (but not *Prosβ2R1*). A statistically significant reduction was seen also with *Ced-6*, *Gαo*, *Twins* (*tws*) and *heph*. Three genes that negatively regulate the Toll pathway were identified: *IntS2*, *IntS6* and *eff*. The effect of *IntS2* and *IntS6* on the Toll pathway has been previously shown (46), but the effect of *eff* on the Toll pathway has not been previously studied and remains to be investigated.

As a summary, the integrator complex members (IntS2 and IntS6) appear to negatively regulate all pathways tested indicating that their effect is not specific to the JAK/STAT pathway. Although Effete co-localizes with ET, it does not seem to regulate the *TotM-luc* reporter gene activation mediated by the JAK/STAT pathway. Instead, Effete appears to negatively regulate the Toll pathway in S2 cells, which requires further exploration in the future. RNAi against the proteasome complex components does not have major effects on the Imd pathway, but silencing proteasome complex members decreases the activity of the Toll pathway indicating that the proteasome positively regulates the Toll pathway. Cactus, the *Drosophila* homologue of the mammalian Inhibitor of κB (IκB), is known to be degraded by the proteasome upon induction of the Toll pathway (47, 48), so it is plausible that silencing components of the proteasome complex leads to accumulation of the Cactus protein and reduction in the activity of the Toll pathway also in this context.

### *Prosα6* Silencing Leads to Reduced Expression of *ET* and Reduced Amounts of the ET Protein

**Figure 2:**
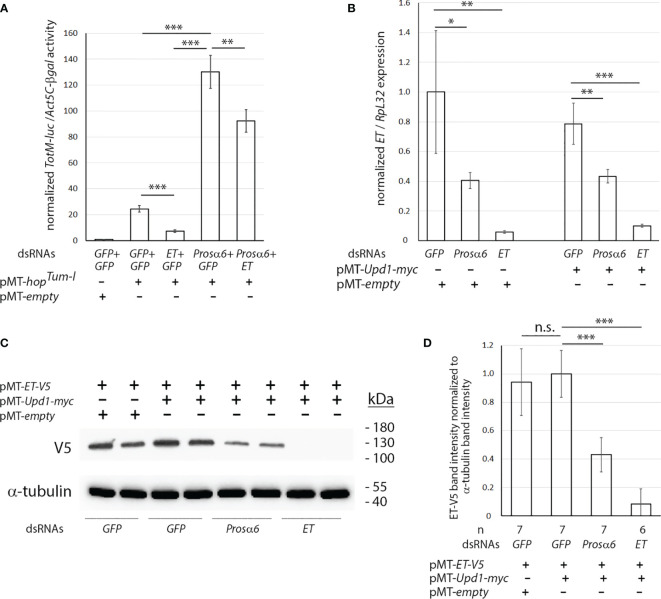
*Prosα6* silencing leads to reduced expression of *ET* and reduced amounts of the ET-V5 protein in S2 cells. **(A)** Double knockdown of *ET* and *Prosα6* by dsRNA treatments in S2 cells had an additive effect to the *hop^Tum-l^* -induced *TotM-luc* activity. **(B)** Knocking down *Prosα6* caused a reduction in *ET* transcription **(C, D)** Knocking down *Prosα6* by dsRNA treatment in S2 cells caused a reduction in the amount of the ET-V5 protein. **(C)** Example of one experiment. **(D)** Quantification of ET-V5 protein bands from three independent experiments, in total six or seven replicates per treatment. *p, 0.05, **p, 0.01, ***p, 0.001. n.s., not significant.

Because the proteasomal genes were identified in JAK/STAT pathway regulation in S2 cells (**Figures 1A, B**), we selected Prosα6 to study in more detail. To study the interplay between ET and Prosα6 in the JAK/STAT pathway, we carried out a double knockdown experiment with *ET* and *Prosα6*. As shown in **Figure 2A**, the *TotM-luc* activity was induced with the *pMT*-*hop^Tum-l^* construct. As previously shown, *ET* knockdown reduces this *hop^Tum-l^* -induced TotM-luc activity [(31) and **Figure 1A**], while *Prosα6* knockdown causes hyperactivation of this activity (**Figure 1A**). When both *ET* and *Prosα6* are knocked down simultaneously (*Prosα6+ET*), the result is additive, indicating that the effect of ET and Prosα6 proteins on the JAK/STAT pathway activation is partially independent.

Next, we investigated what happens to the expression of *ET* upon *Prosα6* knockdown. S2 cells were treated with *Prosα6* dsRNA and control dsRNAs and either transfected with *pMT-Upd1-myc*, which induces the JAK/STAT pathway, or transfected with the *pMT-empty* plasmid for control. *ET* expression values from RNAs extracted from the cells were measured and normalized with *RpL32* expression. As shown in **Figure 2B**, *Prosα6* knockdown causes reduction in the transcription of *ET* both in conditions where the JAK/STAT pathway is inactive, and in those where it is activated with overexpression of *Upd1*.

Further, we investigated the effect of *Prosα6* knockdown on the amount of ET protein, by treating S2 cells with *Prosα6* dsRNA and control dsRNAs and transfecting them with *pMT-ET-V5* together with *pMT-Upd1-myc*, which induces the JAK/STAT pathway. Treatment of cells with the *pMT-empty* vector and *GFP* dsRNA was used as a control. Cellular lysates were prepared and subjected to SDS-PAGE electrophoresis, Western blotting and antibody treatments and imaged. The ET-V5 protein band intensity was normalized to α-tubulin band intensity values in each sample. As shown in **Figures 2C, D**, the amount of ET-V5 protein is reduced in *Prosα6* dsRNA-treated cell lysates compared to the negative control *GFP* dsRNA-treated lysates. **Figure 2C** shows an example of one experiment, and **Figure 2D** shows the quantification from three independent experiments including in total six or seven replicates per treatment. In *ET* dsRNA-treated cells, the ET-V5 band is not at all visible indicating that RNAi in S2 cells silences the protein production of the targeted gene very efficiently.

In conclusion, upon knockdown of *Prosα6*, *ET* transcription as well as the amount of the ET protein is decreased. As ET is the key negative regulator of the JAK/STAT pathway, these results in part explain the hyperactivation of the pathway by knockdown of *Prosα6*.

### *Prosα6* Silencing in Hemocytes Activates JAK/STAT Signaling

**Figure 3:**
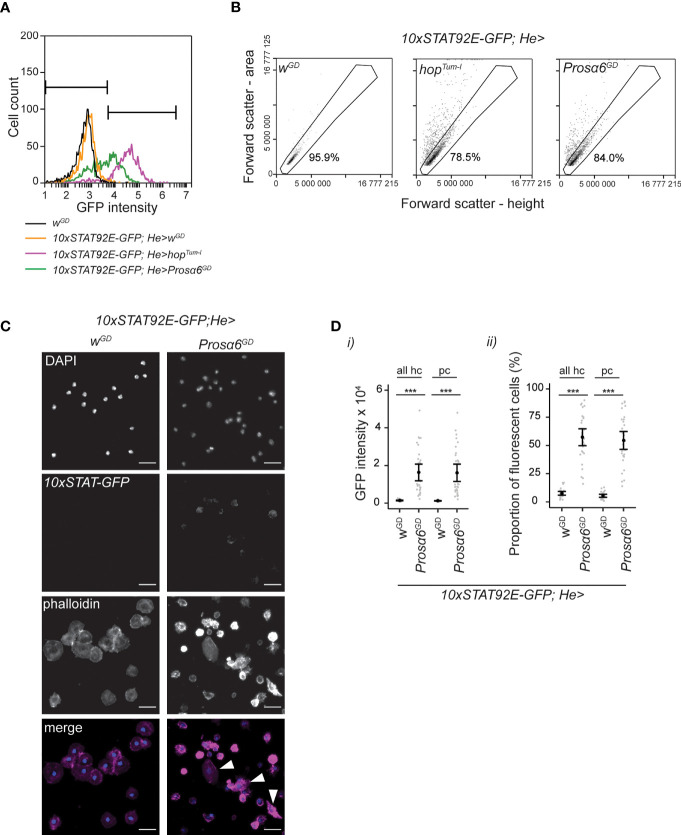
*Prosα6* silencing in hemocytes activates JAK/STAT signaling. **(A)** Examples of GFP fluorescence intensity in non-fluorescent (*w^GD^*, black line), control (orange line), *hop^Tum-l^* overexpressing (purple line) and in *Prosα6* knockdown (green line) hemocytes. Note that in the figures the symbol “>“ denotes the presence of the GAL4/UAS system. The genotypes for control, *hop^Tum-l^* overexpressing and *Prosα6* knockdown animals are *10xSTAT92E-GFP;He-GAL4/w^GD^, 10xSTAT92E-GFP;He-GAL4/UAS-hop^Tum-l^* and *10xSTAT92E-GFP;He-GAL4/UAS-Prosα6^GD^*, respectively. Bars mark GFP-negative (on the left) and GFP-positive (on the right) areas. **(B)** Hemocytes detected in *10xSTAT92E-GFP;He-GAL4/w^GD^*, *10xSTAT92E-GFP;He-GAL4/UAS-hop^Tum-l^* and *10xSTAT92E-GFP;He-GAL4/UAS-Prosα6^GD^* animals in forward scatter area *vs*. height plot. While in the controls (*w^GD^*) most cells resided at a 45° angle in the area *vs.* height plot, the *Prosα6* knockdown and *hop^Tum-l^* overexpressing animals had hemocytes deviating from this area. Gated area represents hemocytes that are considered round (mainly non-activated plasmatocytes). Percentages represent the hemocytes falling into this gate in these example plots. 822 control, 2119 *hop^Tum-l^* overexpression and 1857 *Prosα6* knockdown cells were analyzed for **(A, B)**. **(C)** Fluorescence microscopy of hemocytes from the control and *Prosα6* knockdown larvae expressing the 10xSTAT-GFP and stained with the nuclear stain DAPI and the F-actin stain Phalloidin. Hemocytes with lamellocyte morphology (marked with arrowheads) were found in *Prosα6* knockdown larvae but not in controls. Of note, DAPI staining appears dimmer in the *Prosα6* knockdown sample, but this was likely due to slide-to-slide variation. Scale bars 10 µm. **(D)** Quantification of 10xSTAT-GFP signal intensity and percentage of fluorescent hemocytes in controls and in the *Prosα6* knockdown animals. All hc, the whole hemocyte population; pc, hemocytes inside the gate shown in **(B)**. Hemocytes were analyzed from two replicates of *w^GD^* (nine animals each) and three replicates of *Prosα6^GD^* (10 animals each), comprising of an order of 10^4^ cells in each replicate. Error bars show mean and lower and upper confidence limits (cl). Grey dots represent individual animals. ***p < 0.001. Intensity values were analyzed using a Kruskal-Wallis rank sum test combined with Dunn’s *post hoc* test. The proportions of fluorescent cells were analyzed using a GLM with binomial distribution combined with Tukey’s *post-hoc* test.

Since our previous results indicated involvement of the proteasome complex in regulating JAK/STAT signaling in S2 cells, we next tested if this was also the case *in vivo* in *Drosophila* larval hemocytes. We used a STAT reporter *10xSTAT92E-GFP* combined with a hemocyte driver *He-GAL4* as a read-out of the JAK/STAT activity in hemocytes using flow cytometry. As a positive control we overexpressed a constitutively active *Drosophila* JAK/STAT pathway component *hopscotch* (*hop*) in hemocytes (*10xStat92E-GFP;He-GAL4/UAS-hop^Tum-l^*), and as expected, detected an increase in the *Stat92E-GFP* signal compared to control hemocytes. We also detected a clear increase in the reporter expression when knocking down the *Proteasome α6 subunit* (*10xStat92E-GFP;He-GAL4/UAS-Prosα6^GD^*) compared to control hemocytes expressing only the reporter (**Figure 3A**).

The FSC-area (FSC-A) *vs*. FSC-height (FSC-H) plot is often used to exclude cell doublets from further flow cytometry analysis taking advantage of the fact that round particles will appear as a population at a 45° angle, while doublets have larger area than height. We observed that while the control animals had mainly round hemocytes (plasmatocytes) (49), appearing at a 45° angle in the FSC-A *vs*. FSC-H plot, *Prosα6* silencing resulted in cells detected outside of this area, similar to when *hop^Tum-l^* was overexpressed in hemocytes (**Figure 3B**). Overexpression of *hop^Tum-l^* is known to cause hematopoietic neoplasia leading to the formation of melanotic nodules and a specific hemocyte type, the lamellocyte (6, 42). Furthermore, Avet-Rochex and coworkers (50) identified *Prosα6* (then called *Pros35*) as a suppressor of melanotic nodules in *Drosophila* larval hemocytes. Therefore, we reasoned that at least a fraction of these hemocytes outside the 45° area might be lamellocytes, which, due to their flat and discoidal morphology (49), fall outside the area where the round plasmatocytes are detected in the control animals. Since lamellocytes can be easily separated from plasmatocytes by morphology, we stained hemocytes from *10xStat92E-GFP;He-GAL4/UAS-Prosα6^GD^* and *10xStat92E-GFP;He-GAL4/w^GD^* 3^rd^ instar larvae with the F-actin stain Phalloidin to visualize hemocyte morphology. We found that hemocytes from the *Prosα6* knockdown larvae had more variation in their morphology when compared to the control hemocytes and observed the presence of hemocytes with a lamellocyte morphology (**Figure 3C**).

Next, we analyzed the GFP signal intensity of the *Stat92E-GFP* reporter and the proportion of fluorescent hemocytes separately in all hemocytes and in the plasmatocyte fraction (**Figure 3B**). In both cases, the GFP signal was significantly higher in the hemocytes where *Prosα6* was silenced compared to controls (**Figure 3D**, i). Also, the fraction of GFP-positive hemocytes increased significantly in both populations after *Prosα6* knockdown (**Figure 3D**, ii). These data indicate that silencing *Prosα6* leads to activation of JAK/STAT signaling also *in vivo.*

### Silencing *Prosα6* in Hemocytes Leads to Activation of Hemocytes and Differentiation of an Infection-Induced Hemocyte Type, the Lamellocyte

**Figure 4:**
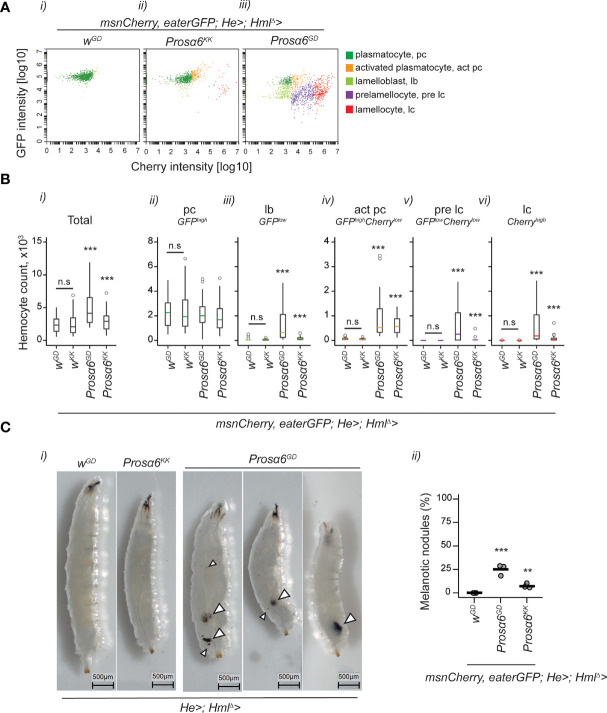
*Prosα6* silencing in hemocytes leads to an increase in total hemocyte numbers and to the formation of activated hemocytes. **(A)** Examples of flow cytometry plots showing hemocytes expressing *eaterGFP* (a plasmatocyte marker) and *msnCherry* (a lamellocyte marker). A wild-type larva had mainly *eaterGFP*-positive plasmatocytes *(i)*, whereas *Prosα6* silencing in hemocytes (*msnCherry,eaterGFP;Hml^Δ^-GAL4;He*-*GAL4/UAS-Prosα6*) resulted in the formation of lamellocytes (*ii*) or a full-blown activation of hemocytes (*iii*). **(B)** Quantification of total hemocytes (i) and each hemocyte class (*ii-vi*) from control (*msnCherry,eaterGFP;Hml^Δ^-GAL4; He*-*GAL4/w^GD^* and *msnCherry,eaterGFP;Hml^Δ^-GAL4; He*-*GAL4/w^KK^*) larvae and from larvae with *Prosα6* silencing in hemocytes (*msnCherry,eaterGFP;Hml^Δ^-GAL4; He*-*GAL4/UAS-Prosα6* ^GD^ and *msnCherry,eaterGFP;Hml^Δ^-GAL4; He*-*GAL4/UAS-Prosα6* ^KK^). Each genotype was replicated three times, 10 animals in each replicate. Error bars show mean and lower and upper 95% confidence limits (cl). Grey dots represent individual animals. Data on hemocytes were analyzed using a negative binomial GLM. Stars indicate a significant difference when compared to the control sample. The two control backgrounds were also compared to each other. n.s., not significant; ***p < 0.001; pc, plasmatocyte; act pc, activated plasmatocyte; lb, lamelloblast; pre lc, prelamellocyte; lc, lamellocyte. **(C)**, *i*) Examples of larvae without and with melanotic hemocyte aggregates of varying sizes, some of which are marked with arrowheads. For nodule quantification, each genotype was replicated three times, with 100 animals in each replicate (ii). Data were analyzed as a proportion of animals bearing nodules, using a GLM with binomial distribution combined with Tukey’s *post-hoc* test. ***p < 0.001; **p < 0.01.

Prompted by the observation of the appearance of lamellocytes and JAK/STAT reporter activation after *Prosα6* silencing in hemocytes, we studied the hemocyte phenotype further. To investigate if silencing of *Prosα6* in hemocytes causes hemocyte differentiation, we utilized hemocyte reporter constructs *eaterGFP* (expressed in plasmatocytes) and *msnCherry* (expressed in lamellocytes) combined with the *Hml^Δ^-GAL4* and *He-GAL4* to silence *Prosα6* in hemocytes and to quantify hemocyte types using a flow cytometer. Flow cytometry analysis of hemocytes showed that while in a wild-type larva, the majority of the hemocyte pool consists of *eaterGFP*-positive plasmatocytes, the silencing of *Prosα6* caused aberrant lamellocyte differentiation and hemocyte activation (**Figure 4A**, *i-iii*). The hemocyte response was stronger with the *UAS-Prosα6^GD^* construct, where the numbers of all identified hemocyte types were increased, including lamelloblasts, the putative lamellocyte precursors, and prelamellocytes (**Figure 4B**, *i-vi*). A similar, but milder, response was seen also with the *UAS-Prosα6^KK^* construct. Furthermore, around 25% of *Hml^Δ^-GAL4;He-GAL4/UAS-Prosα6^GD^* larvae exhibited melanotic nodules, while in *Hml^Δ^ -GAL4;He-GAL4/UAS-Prosα6^KK^* larvae this phenotype was again milder (**Figure 4C**, *i-ii*).

Since silencing *Prosα6* in hemocytes led to hemocyte activation and melanotic nodule formation at the larval stage, we further studied the effects of *Prosα6* silencing on egg-to-adult development and the lifespan of the flies. We found that while egg-to-pupal development of the animals with *Prosα6* silencing in hemocytes was comparable to the controls (**Supplementary Figure 2A**), the pupal eclosion rate was lower in animals expressing *UAS-Prosα6^GD^* in hemocytes, whereas animals expressing *UAS-Prosα6^KK^* eclosed at a normal rate (**Supplementary Figure 2A**). Inspection of the pupal cases revealed melanotic nodules in a fraction of animals with *Prosα6* silencing (1% in *msnCherry,eaterGFP;Hml^Δ^-GAL4;He*-*GAL4/*/*UAS-Prosα6^KK^* and 20% in *msnCherry,eaterGFP;Hml^Δ^-GAL4;He*-*GAL4/*/*UAS-Prosα6^GD^;* **Supplementary Figure 2B**), similar to the levels observed in larvae (**Figure 4C**). The lifespan of the *msnCherry,eaterGFP;Hml^Δ^-GAL4;He*-*GAL4/UAS-Prosα6^GD^* flies, both males and females, was shorter than that of the control flies, whereas the lifespan of *msnCherry,eaterGFP;Hml^Δ^-GAL4;He*-*GAL4/UAS-Prosα6^KK^* flies was not affected (**Supplementary Figures 2C, D**). When the expression of *Prosα6* normalized to *RpL32* was measured from hemocytes by qRT-PCR, it was shown that in the *Hml^Δ^-GAL4;He-GAL4*/*UAS-Prosα6^GD^* hemocytes, *Prosα6* expression was 43 ± 14% from the respective control (*w*/*Prosα6^GD^*), whereas in the *Hml^Δ^-GAL4;He-GAL4*/*UAS-Prosα6^KK^* hemocytes, *Prosα6* expression was 52 ± 17% from the control (*w/UAS-Prosα6^KK^*) hemocytes (**Supplementary Figure 2E**).

To conclude, knocking down *Prosα6* in hemocytes causes immune activation characterized by the differentiation and activation of hemocytes and the formation of melanotic nodules, but also decreases the viability of the flies. It is possible that there is a threshold level of silencing needed for the full phenotypic effects, as with the KK line, only the mild activation of hemocytes, but not the other phenotypes, is seen.

### JAK/STAT and JNK Signaling Are Needed for the Full Hemocyte Activation Caused by *Prosα6* Silencing

**Figure 5:**
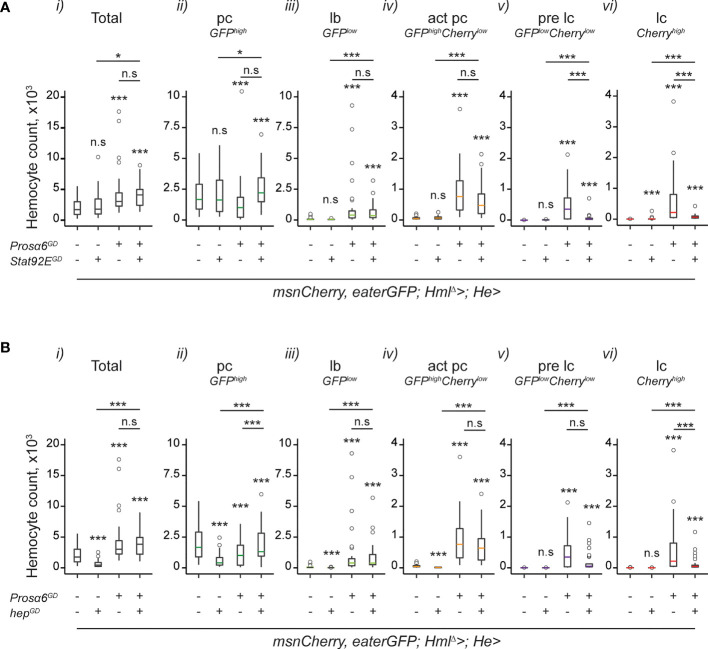
Knocking down JAK/STAT and JNK pathway components in the *Prosα6* background reduce the hemocyte activation caused by the *Prosα6* silencing. **(A)** Effects of JAK/STAT pathway component *Stat92E* knockdown on *Prosα6*-induced hemocyte phenotype. i) Total hemocyte counts. *ii-vi*) Differential hemocyte counts. “-/+” indicate the presence of *Prosα6* and *Stat92E* knockdowns. Stars refer to the statistical difference compared to the wild-type control larvae (*msnCherry,eaterGFP;Hml^Δ^-GAL4;He*-*GAL4/w^GD^)*, the first sample in each plot. Underlined stars refer to the statistical difference of the simultaneous knockdown of *Prosα6* and *Stat92E* (*msnCherry,eaterGFP;Hml^Δ^-GAL4;He*-*GAL4/UAS- Prosα6^GD^;UAS-Stat92E^GD^*) to the single knockdowns of *Prosα6* (short line) and *Stat92E* (longer line). **(B)** Effects of JNK pathway component *hep* knockdown on *Prosα6*-induced hemocyte phenotype. i) Total hemocyte counts. *ii-vi*) Differential hemocyte counts. “-/+” indicate the presence of *Prosα6* and *hep* knockdowns. Stars refer to the statistical difference compared to the wild-type control larvae (*msnCherry,eaterGFP;Hml^Δ^-GAL4;He*-*GAL4/w^GD^*), the first sample in each plot. Underlined stars refer to the statistical difference of the simultaneous knockdown of *Prosα6* and *hep* (*msnCherry,eaterGFP;Hml^Δ^-GAL4;He*-*GAL4/UAS- Prosα6^GD^;UAS-hep^GD^*) to the single knockdowns of *Prosα6* (short line) and *hep* (longer line). Each genotype was replicated three times, except for the *msnCherry,eaterGFP;Hml^Δ^-GAL4;He*-*GAL4/w^GD^* control, which was replicated 6 times, 10 animals in each replicate. Note that the control and *Prosα6* hemocyte data is the same in **(A, B)** and has been plotted separately for clarity. Statistical analyses have been conducted on the whole dataset and p-values adjusted according to multiple comparisons. Data was analyzed using a negative binomial GLM. n.s., not significant; *p < 0.05; ***p < 0.001; pc, plasmatocyte; act pc, activated plasmatocyte; lb, lamelloblast; pre lc, prelamellocyte; lc, lamellocyte.

To further verify that *Prosα6* silencing induces hemocyte activation *via* JAK/STAT signaling, we opted for a genetic rescue experiment. We knocked down *Prosα6* using the GD construct simultaneously with *Stat92E* and checked the effect on the hemocyte phenotype. Since also the JNK pathway has been shown to be important in lamellocyte formation (6), we also tested whether it has a role in hemocyte activation in the *Prosα6* silencing background. To this end, we knocked down *Prosα6* simultaneously with *hemipterous* (*hep*), a *Drosophila* JNK-pathway component and checked the hemocyte composition. Knocking down *Stat92E* alone in hemocytes resulted in a mild activating effect; a small number of lamelloblasts and lamellocytes were detected in an otherwise wild type background (**Figure 5A**). Knocking down *hep* led to a reduction in total hemocyte numbers compared to the control (**Figure 5B**). Neither *Stat92E* nor *hep* knockdown in the *Prosα6*-silenced background led to the rescue of total hemocytes, or of plasmatocytes, lamelloblasts or activated plasmatocytes, back to the levels detected in the control animals (**Figures 5A, B**, i-iv). Both, however, reduced the formation of prelamellocytes and lamellocytes induced by *Prosα6* silencing (**Figures 5A, B**, v-vi). Even though knocking down neither *Stat92E* nor *hep* in the *Prosα6* background rescued the hemocyte phenotype completely to the wild-type levels, the number of lamellocytes produced by the *Prosα6* knockdown (600 on average) was reduced approximately seven-fold with *Stat92E* knock-down (51) and four-fold with *hep* knockdown (148). Taken together, these results suggest that *Prosα6* silencing-induced hemocyte activation requires the function of the JAK/STAT pathway, and that the JNK pathway functions in parallel or downstream of JAK/STAT eliciting hemocyte activation.

## Discussion

In this study we have investigated the potential interaction partners of ET, and other putative regulators of the JAK/STAT pathway using our previously unpublished RNAi screen data. Among the 25 genes that were selected for further study, 12 have a described function in the proteasome pathway. These were *Rpn1*, *Rpn2*, *Rpn3*, *Prosα1*, *Prosα6*, *Prosα7*, *Prosβ2*, *Prosβ3*, *Prosβ5*, *Prosβ7*, *Prosβ2R1* and *Pomp*, all of which caused elevation of *TotM*-luc activity upon activation of the JAK/STAT pathway by *hop^Tum-l^* in S2 cells. The 26S proteasome is a large complex composed of many subunits and under normal conditions, it degrades most proteins in the cell (52). It is known that the half-life of a protein varies from minutes to months, but at some point, every protein is marked and brought to degradation. In eukaryotes, unwanted proteins are marked for proteasomal degradation mainly with K48-linked polyubiquitin chains (53), which are recognized by the proteasome 19S regulatory particle. The regulatory particle functions in binding the ubiquitinated target proteins and deubiquitinating, unfolding and translocating them to the 20S proteasome core particle for cleavage (43). The core particle is a barrel shaped structure containing two outer rings each formed by seven α-subunits, and two inner rings formed by seven β-subunits. The β-subunits create a chamber where the proteolytically active sites are located. The N-termini of α-subunits form a gate that lets the substrates (target proteins) in through the central α-ring channel (52).

The ubiquitin-proteasome pathway has been implicated in the regulation of the JAK/STAT pathway in mammals: treatment with proteasome inhibitors prolongs the activation of the JAK/STAT pathway in response of many cytokine stimuli, e.g (54–56). The mechanisms of proteosome-related regulation of the JAK/STAT pathway are complex: treatment with proteasome inhibitors causes stabilization of tyrosine-phosphorylated forms of STAT1 (56), STAT4, STAT5, STAT6 (57) and Jak2 (58), and thereby prolonged JAK/STAT activation. Furthermore, treatment with proteasome inhibitors also causes stabilization of the complex of the Jak2 protein with SOCS-1 (Suppressor Of Cytokine Signaling-1); SOCS-1 expression would normally enhance the proteasomal degradation of Jak2, but when the proteasome function is blocked, the Jak2/SOCS-1 complexes accumulate (58). So in mammals, the SOCS proteins (eight proteins, SOCS-1-7 and CIS) (59) bring another layer to the regulation of the JAK/STAT pathway. The *Drosophila* JAK/STAT pathway has similar core pathway components as mammals, but there is less redundancy. For instance, in *Drosophila*, three members of the SOCS family have been identified, out of which Socs36E appears to be the main negative regulator of the pathway (25, 60). Similarities have been found also in the regulation of the *Drosophila* and mammalian JAK/STAT pathways, such as between the *Drosophila* non-signaling receptor/inhibitor ET and the mammalian gp130, the soluble form of which can inhibit signaling (25, 31, 61).

We show that the knockdown of the *Proteasome α6 subunit* results in reduction in *ET* transcription, and reduction in the amount of the ET protein in *Drosophila* S2 cells (31). It appears that *Prosα6* expression is needed for full *ET* expression to prevent aberrant activation of the JAK/STAT pathway. This regulation may be very complex: for example, Prosα6 knockdown, i.e., inhibition of the normal proteasome function, may cause an accumulation of other regulatory protein(s) causing a reduction in *ET* expression. The reduction mechanism appears to inhibit ET production already at the transcriptional level, and this effect is seen in both basal conditions and in conditions, where the JAK/STAT pathway is activated.

Moreover, we show that silencing *Prosα6* in *Drosophila* hemocytes *in vivo* and thereby blocking the proteasomal degradation/turn-over step leads to overactivation of JAK/STAT signaling, aberrant activation of immune cells and the formation of clusters of activated immune cells, known as melanotic nodules or pseudotumors. Our findings are in line with an earlier *in vivo* RNAi screen conducted by Avet-Rochex & coworkers (50), who looked for genes involved in blood cell homeostasis in *Drosophila*. In the screen, they identified *Prosα6* (*Pros35*) as a suppressor of melanotic nodules in hemocytes. Moreover, we show that there is partial rescue of the *Prosα6* knockdown-induced hemocyte activation phenotype not only with knockdown of *STAT92E*, but also *hemipterous* (*hep*), the Jun-kinase in *Drosophila*. These results may indicate that the pathways act synergistically, both being needed for full hemocyte activation. For example, the JNK and JAK/STAT pathways act in cooperation in the wing imaginal disc resulting in the loss of cell fate specification as a response to damage (62). Alternatively, it has been shown that the JAK/STAT ligands *upd1-3* are transcriptional targets of the JNK pathway (63). Therefore, it is possible that the silencing of the JNK pathway leads to reduced *upd* expression, and hence has a dampening effect on the overall activity of the JAK/STAT signaling in hemocytes. So, in addition to the JAK/STAT pathway activation, the JNK pathway seems to be needed for full blown hemocyte activation brought upon by *Prosα6* silencing, but the exact mechanism of this interaction remains to be elucidated.

The proteasome has also been shown to be involved in the turn-over and regulation of several key proteins in immune signaling. On the one hand, proteasomal degradation of inhibitory molecules such as the Inhibitor of κB (IκB) is essential for pathway activation (64) but on the other hand, shutting down un-needed immune activation is equally important, and proteasomal degradation of key factors is one such regulatory mechanism (58, 65). In *Drosophila*, it has been shown that the proteasome represses the Imd pathway, probably by causing the degradation of Relish (NF-kB factor in the *Drosophila* Imd pathway) (66). Here we show that the proteasome is needed for proper Toll pathway activity, likely because Cactus (*Drosophila* IκB in the Toll pathway) has to be degraded upon pathway activation (47, 48).

In addition to proteasome-related genes, we identified additional genes that interact with ET and/or affect the JAK/STAT activity (*Gαo*, *Gαi, Mlc-c*, *Moe*, *tws*, *eff*, *Fur, heph*, *Kap-α3, IntS2* and *IntS6*). Importantly, the effect of a specific knockdown was dependent on the means of the pathway activation. Silencing of two out of eight genes studied with both inducers (Upd1 and Hop^Tum-l^) resulted in a change on the *TotM-luc* reporter expression with both inducers: *Mlc-c* (to the same direction) and *tws* (to opposite directions). Mlc-c forms an essential light chain of Non-muscle myosin II, which is involved in shaping the actin cytoskeleton of cells in e.g., development (67). Since JAK/STAT functions in the control of myogenic differentiation (68), it might be that in this case the activation of the signaling pathway is in response to reduced levels of myosin II. *Drosophila tws* encodes a regulatory B/PR55 subunit of protein phosphatase 2A (PP2A), and it has been shown to play a role in e.g., cell division, tissue patterning and multiple signaling pathways (69, 70). In human T-cell lines, inhibition of PPA2 has been shown to attenuate at least STAT3, STAT5 and STAT6 function (71–73), and there is some indication that it may act similarly in *Drosophila* neuroblasts (74).

Silencing of all the rest of the genes appeared to affect the JAK/STAT signaling activity only in a specific induction context. It is also possible that in some cases, the lack of significant effect may be due to insufficient silencing of the corresponding genes, since in this screen for putative interactors, we only tested one dsRNA per gene. Gαo and Gαi belong to the conserved family of the heterotrimeric G protein α subunits, which act as effector molecules of G-protein coupled receptors (GPCRs) (75). Both Gαo and Gαi were found to activate STAT3 in a murine fibroblast cell line (76, 77), whereas we found that knockdown of Gαo enhanced the Upd1-induced JAK/STAT pathway activity and knock-down of Gαi reduced the Hop^Tum-l^ -induced JAK/STAT. Moesin is a member of the FERM domain (band 4.1, Ezrin, Radixin and Moesin), which is also one of the protein domains at the N-terminus of Jaks enabling the adaptor and scaffolding interactions (78, 79). Similarly in *Drosophila*, moesin is a member of the FERM protein domain, and is an important factor in processes including cell adhesion, cell movement and membrane trafficking (51).

The ubiquitin-conjugating enzymes function in mediating a variety of ubiquitin modifications, including the K48-linked polyubiquitination that directs the ubiquitinated proteins for degradation by the 26S proteasome (80) as well as K63-linked activating polyubiquitination events (45, 81). Eff is an E2 ubiquitin-conjugating enzyme, and it has previously been shown to be needed for the K63-linked polyubiquitination of the cleaved Imd molecule for functional Imd signaling (45). Similarly to the Imd pathway, *eff* knockdown has a positive regulatory effect on the Hop^Tum-l^ -induced JAK/STAT pathway in S2 cells. On the other hand, our results indicate that Eff negatively regulates the Toll pathway in S2 cells, but it appears not to be needed in the fat body mediated defense *in vivo*. Furins are a family of evolutionarily conserved serine endoprotease enzymes that cleave precursor proteins into their mature forms (82). In accordance with our results, silencing of *Drosophila Fur1* in the fat body has previously been shown to elevate the transcription of stress response genes including the JAK/STAT pathway target gene *TotM* (83).

The Notch signaling pathway is well conserved from flies to humans and has been shown to be essential for blood cell development and lineage speciation (11, 84). Kap-α3 (also called importin-α3) has been shown to be required for regulating Notch signaling (85) and Heph has been shown to be an important protein regulating Notch signaling in wing development (86). Both Kap-α3 and Heph negatively regulate the Hop^Tum-l^ -induced JAK/STAT pathway in S2 cells indicating interplay between the Notch and JAK/STAT signaling pathways in this context. The integrator complex genes, in turn, have been suggested to have multiple functions: the main function of the complex is to mediate the 3’ processing of small nuclear RNAs (87). In addition, silencing of the zebrafish Integrator 5 or 11 has been shown to result in defects in hematopoiesis; this was suggested to be due to misprocessing of snRNA, which leads to splicing defects in the mRNA of genes required for hematopoiesis (88). We show that silencing of *IntS2* and *IntS6*, members of the *Drosophila* Integrator complex, results in an increase in the activity of the JAK/STAT pathway. The integrator complex has previously also been shown to negatively regulate the Toll pathway (46).

We also identified proteins that did interact with ET but did not affect JAK/STAT activity in our S2 assay (*Rala* and *ced-6*). Rala, which belongs to the family of Ras-like (Ral) small GTPases homologous to Gα-proteins, is shown to be required cell-autonomously in regulating polar-cell specific markers, including the JAK/STAT pathway ligand Upd, in the developing oocyte (89). Although associating in the complex with ET, it appears that in the S2-cell context, *Rala* knockdown affects neither the Upd1 nor the Hop^Tum-l^ -induced activity of the JAK/STAT pathway. Ced-6 has been identified as an essential adaptor protein for apoptotic clearance of unneeded cells by hemocytes in the developing embryo (90) as well as phagocytosis of *S. aureus* gram-positive bacteria in *Drosophila* adults upon infection (91). It has not previously been implicated in the regulation of JAK/STAT signaling to our knowledge.

The JAK/STAT signaling pathway, like many other signaling pathways, has core and tissue/cell type specific signaling outcomes (92, 93). Accordingly, also the regulatory events outside the core pathway components might vary among the cell types. Our data revealed several putative JAK/STAT regulators in S2 cells, whose mode of action was often dependent on the way the pathway was activated. Importantly, silencing of the proteasome complex member *Prosα6* consistently enhanced the JAK/STAT pathway activity. Moreover, knockdown of *Prosα6* is sufficient to induce the activation of *Drosophila* blood cells, the hemocytes, *in vivo*, *via* a JAK/STAT pathway-dependent mechanism. The interplay between the *Drosophila* JAK/STAT pathway components and negative regulators including ET and the proteasome appears complex and remains to be further explored in detail.

## Data Availability Statement

The original contributions presented in the study are included in the article/**Supplementary Material**. Further inquiries can be directed to the corresponding author.

## Author Contributions

MJ-S, LV, MR, and SV designed the experiments. MJ-S, MM, JC, and SV carried out the *in vitro* S2 cell experiments. LV, MJ-S, and SV performed the *in vivo* larval hemocyte experiments. All authors analyzed their own data. LV, MJ-S, MR, and SV wrote the paper. All authors contributed to the article and approved the submitted version.

## Funding

This work was supported by the Tampere University Doctoral Programme in Medicine and Life Sciences and The Finnish Cultural Foundation (to MJ-S); The Sigrid Juselius Foundation, the Academy of Finland (Grant 277495), the Competitive State Research Financing of the Expert Responsibility Area of Oulu University Hospital and the Tampere Tuberculosis Foundation (to MR); and the Tuberculosis foundation (to SV). The Tampere *Drosophila* Facility, the Tampere Imaging Facility and the Tampere Facility of Flow Cytometry are all partially funded by Biocenter Finland.

## Conflict of Interest

The authors declare that the research was conducted in the absence of any commercial or financial relationships that could be construed as a potential conflict of interest.

## Publisher’s Note

All claims expressed in this article are solely those of the authors and do not necessarily represent those of their affiliated organizations, or those of the publisher, the editors and the reviewers. Any product that may be evaluated in this article, or claim that may be made by its manufacturer, is not guaranteed or endorsed by the publisher.

## Abbreviations

AMPs, Antimicrobial peptides; dsRNA, double-stranded RNA; GFP, Green Fluorescent Protein; hop^Tum-l,^ Hopscotch Tumorous-lethal; He, hemese; hep, hemipterous; Hml, Hemolectin; imd, Immune Deficiency; JAK, Janus kinase; JNK, c-Jun terminal kinase; STAT, Signal Transducer Activator of Transcription; pMT, plasmid with a metallothionein promoter; RNAi, RNA interference; TotM, Turandot M; UAS, Upstream Activating Sequence; upd, Unpaired.
