# Supplementary material for: Proteasome α6 Subunit Negatively Regulates the JAK/STAT Pathway and Blood Cell Activation in Drosophila melanogaster
Source: Front Immunol. 2021 Dec 22;12:729631. doi: 10.3389/fimmu.2021.729631 (PMC8727353; doi:10.3389/fimmu.2021.729631)
Supplement: Supplementary file 1 [file DataSheet_1.docx]

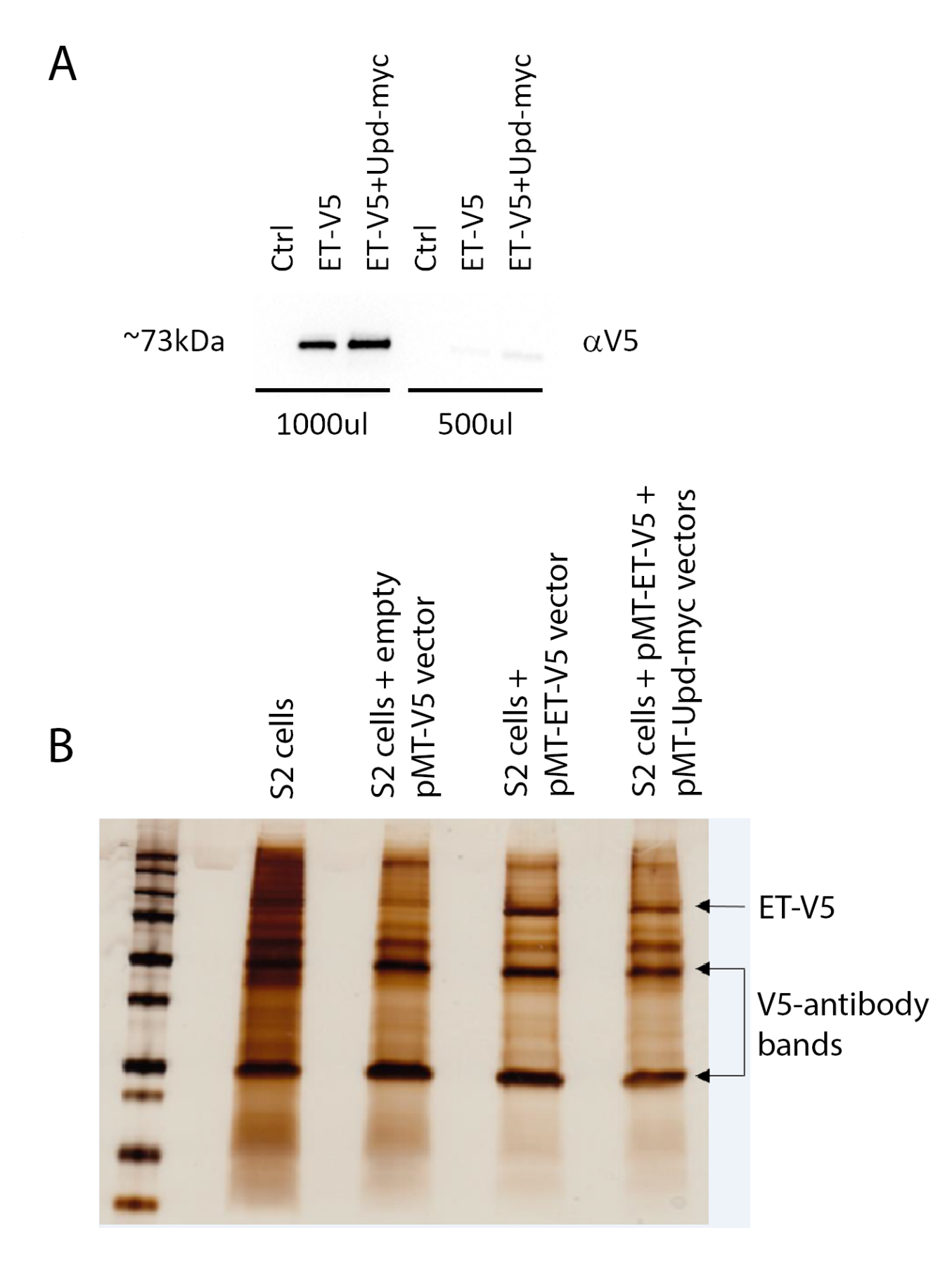


**Supplemental Figure 1.** Identification of putative interaction partners of Eye transformer (ET). A) Western blot with the α-V5 antibody shows that the ET-V5 signal is clearly visible in the protein lysates with ET-V5 overexpression. B) Silver staining of affinity-purified samples.
