# Supplementary material for: Proteasome α6 Subunit Negatively Regulates the JAK/STAT Pathway and Blood Cell Activation in Drosophila melanogaster
Source: Front Immunol. 2021 Dec 22;12:729631. doi: 10.3389/fimmu.2021.729631 (PMC8727353; doi:10.3389/fimmu.2021.729631)
Supplement: Supplementary file 2 [file DataSheet_2.docx]

**
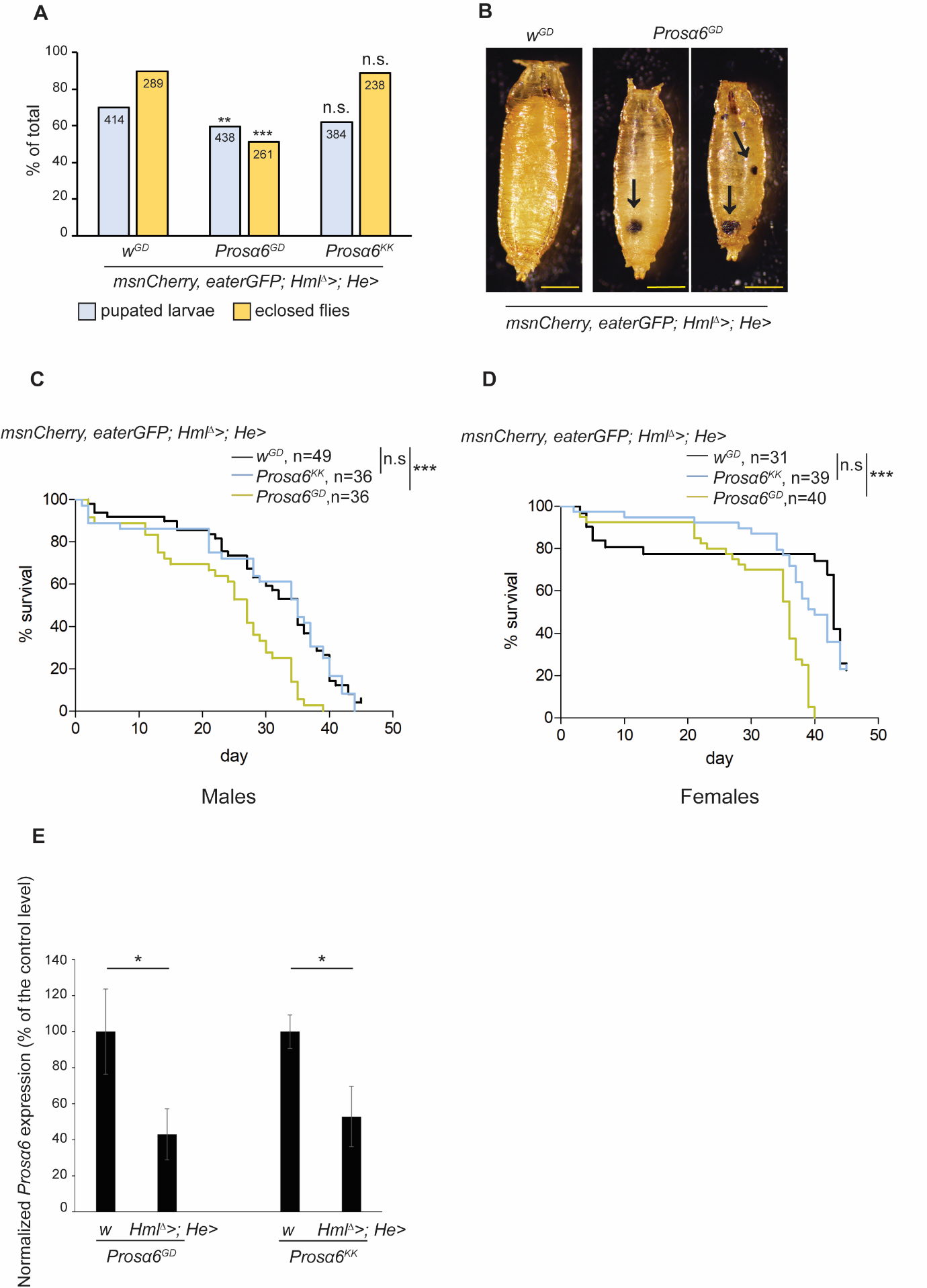
**

**Supplemental Figure 2. Knockdown of *Prosα6*, but not *Prosα6^KK^*, in hemocytes reduces the viability of the flies.** A) Proportion of successfully pupated larvae and eclosed flies in the control (*msnCherry,eaterGFP;Hml^Δ^-GAL4;He*-*GAL4/w^GD^*) and *Prosα6* knockdown (*msnCherry,eaterGFP;Hml^Δ^-GAL4;He*-*GAL4/UAS-Prosα6*) animals. Numbers inside the bars indicate the total amount of eggs (in blue columns) and total amount of pupae (in yellow columns). Data was analyzed using a GLM with binomial distribution combined with Tukey’s post-hoc test. P-values indicate the difference to the *w^GD^* control. B) Examples of pupae with nodules. C-D) Lifespan of *w^GD^* control and *Prosα6* knock-down males (C) and females (D). Statistical analyses of fly life span experiments were carried out with the log-rank (Mantel-Cox) test using Prism 6 (GraphPad) software. E) Expression level of *Prosα6* when silenced (*Hml^Δ^-GAL4;He*-*GAL4/UAS-Prosα6*) with the two RNAi constructs used in the experiments (*UAS-Prosα6^GD^*  and *UAS-Prosα6^KK^*) presented as a percentage of that in the control (w/ *UAS-Prosα6*) samples. Error bars present the standard deviation. Pairwise 2-tailed Student’s t-test was used to analyze the data using the 2ΔCt values. ***, p<0.001; **, p<0.01; *, p< 0.05; n.s., not significant.
